# Secretory leukocyte protease inhibitor (SLPI) as a potential target for inhibiting metastasis of triple-negative breast cancers

**DOI:** 10.18632/oncotarget.22660

**Published:** 2017-11-26

**Authors:** Sergey V. Kozin, Nir Maimon, Rong Wang, Nisha Gupta, Lance Munn, Rakesh K. Jain, Igor Garkavtsev

**Affiliations:** ^1^ Department of Radiation Oncology, Edwin L. Steele Laboratory for Tumor Biology, Massachusetts General Hospital, Boston, MA, USA

**Keywords:** SLPI, triple-negative breast cancer, FoxM1, anti-metastatic compound

## Abstract

SLPI has been implicated in the progression and metastasis of certain cancers. However, the effects of SLPI seem to be tumor-specific and the mechanisms remain poorly defined. Here, we demonstrate that highly metastatic, triple-negative breast cancer (TNBC) 4T1 cells secreted more SLPI compared to their non-metastatic counterparts. Furthermore, SLPI secretion directly correlated with spontaneous lung metastasis from 4T1 tumors orthotopically implanted in mice. Consistent with our experimental results, we also found that higher SLPI expression levels correlate with worse clinical outcome in basal/TNBC patients. Using high-throughput screening we identified a novel compound, C74, which significantly inhibits SLPI secretion. C74 administration in our mouse model slows the growth of primary 4T1 tumors and inhibits their dissemination to the lung. We also discovered that SLPI physically interacts with the retinoblastoma tumor suppressor protein (Rb) and releases FoxM1 from the Rb-FoxM1 complex, which may activate FoxM1 target genes involved in breast cancer metastasis.

## INTRODUCTION

Triple negative breast cancer (TNBC) is the most aggressive breast cancer subtype and has the worst prognosis, despite advancements in modern therapeutics [1]. Drug resistance, tumor invasion and metastasis [2] all contribute to the poor prognosis of TNBC, and better targeted therapies and more effective systemic treatments are urgently needed. There are some indications that breast cancer cells secrete substances that facilitate dissemination, including osteopontin, hyaluronan, metalloproteases and integrin-binding ligands [3, 4]. However, it is not clear what role these components play during invasion and metastasis of TNBC, or whether they are valid targets for inhibiting metastasis in patients [5].

SLPI is a secreted serine protease inhibitor serving as an important protective component of the mucosa and skin [6, 7]. The function of SLPI has been the subject of extensive investigation since it exerts pleiotropic activities in multiple biological contexts. SLPI is known to be involved in the inhibition of inflammation, the modulation of immunological responses and the promotion of cell proliferation [7-9]. In cancer, there is a body of literature showing increased SLPI expression not only in breast cancer, but also in lung, gastric and colorectal carcinomas [10-13]. For example, patients with non-small cell lung carcinoma have higher serum SLPI levels than patients with small cell lung carcinoma or healthy individuals, and these levels were found to correlate with tumor stage [10]. However, in some cases overexpression of SLPI at the primary tumor site repressed metastases [14-15]. In breast cancer, overexpression of SLPI is usually associated with more aggressive, metastatic disease [16-19]. However, the details of how SLPI influences tumor aggressiveness, metastatic potential and treatment outcome remain unknown.

Here, we show that highly metastatic murine 4T1 TNBC breast cancer cells produce higher levels of SLPI compared to their non-metastatic 67NR counterparts and that SLPI level is associated with increased lung metastasis from orthotopically implanted 4T1 tumors. Applying a high-throughput screening assay to a small-molecule library, we selected a compound that represses SLPI pharmacologically and decreases metastasis to the lung. We identified two possible mechanism by which SLPI promote metastasis. First, we found overexpression of SLPI facilitated breast cancer cell invasion of an endothelial monolayer. And second, we found that SLPI physically interacts with and phosphorylates retinoblastoma (Rb) tumor suppressor protein, releasing Forkhead box transcriptional factor M1 (FoxM1) from Rb-FoxM1 complex to activate FoxM1 target genes. FoxM1 is known to be up-regulated in the majority of solid human cancers, including TNBC breast cancer, and is implicated in invasion and metastasis [20-23]. Thus, we propose that SLPI may be a new target for anti-metastatic therapies for TNBC.

## RESULTS

### SLPI secretion is increased in highly metastatic cancer cells and promotes lung metastasis

**Figure 1:**
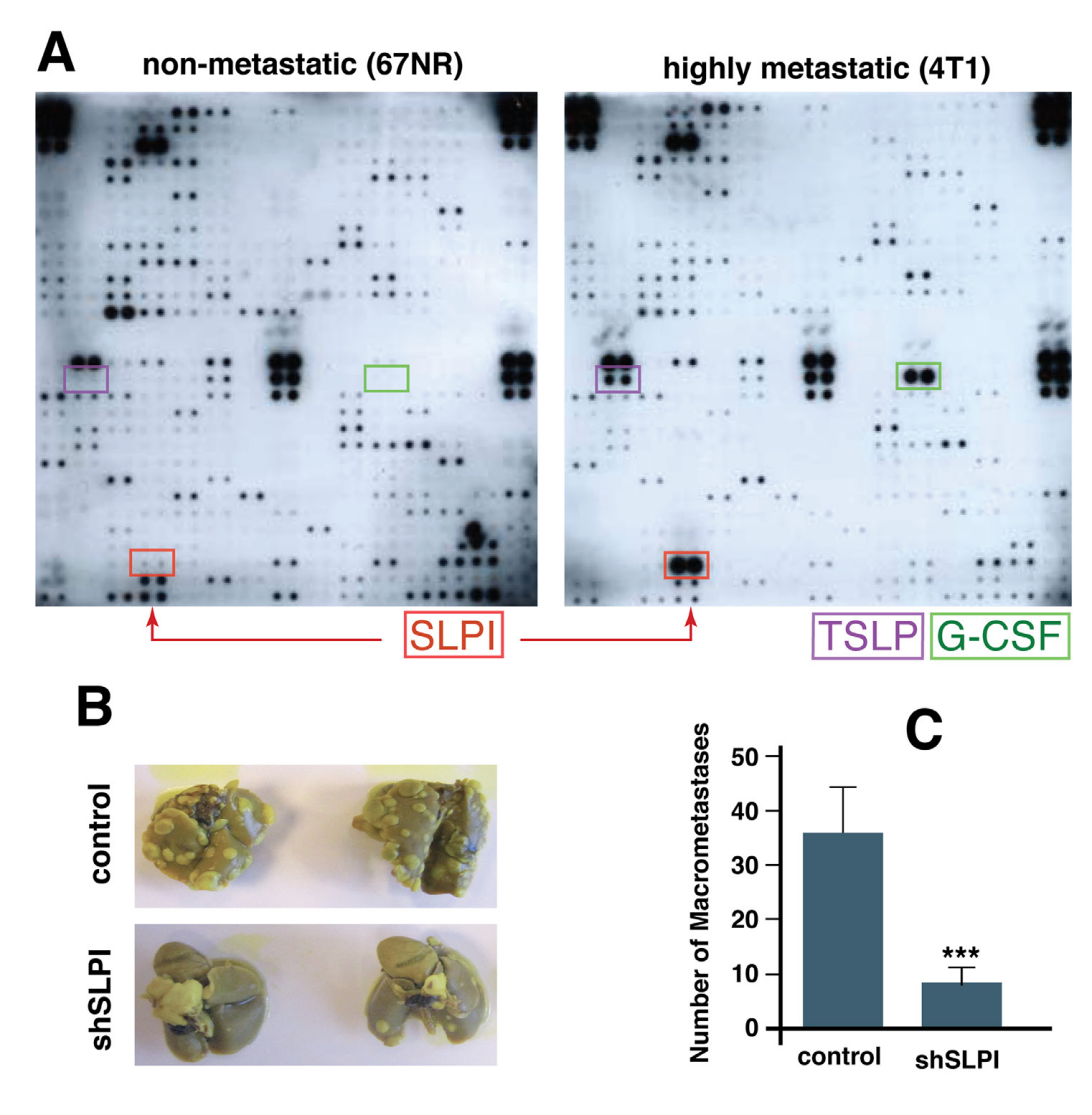
SLPI is upregulated in highly metastatic cancer cells and promotes spontaneous lung metastasis in mice **A.** Comparison of secreted proteins in non-metastatic (67NR) and highly metastatic (4T1) cell conditioned media using a protein array that detects 350 mouse proteins. Arrows indicate secreted proteins markedly increased in highly metastatic cells including SLPI. **B.** Down-regulation of SLPI using shRNA in 4T1 cells inhibits lung metastases. The mice were implanted with control or SLPI-shRNA 4T1 tumors orthotopically and the lungs were examined for metastases (whitish nodules) 21 days after primary tumor resection. Lungs from mice pre-implanted with SLPI-shRNA tumors clearly exhibited fewer metastases (shSLPI). **C.** The number of macrometastases (colonies at least 0.5 mm in diameter) per mouse was significantly less in the mice bearing the SLPI-shRNA tumors (*n* = 7 for each group, *p = 0.004).

First, to assess if highly metastatic murine breast cancer cells (4T1) secrete different factors from those secreted by strain-matched but non-metastatic cells (67NR), we screened conditioned media from both cell lines using a mouse-specific antibody array. We found three secreted proteins that were selectively and significantly increased in 4T1 cells: SLPI, TSLP (thymic stromal lymphopoietin), and G-CSF (granulocyte colony-stimulating factor) (Figure 1A). To determine if the difference in SLPI secretion was unique to these TNBCs, we also analyzed conditioned media from a highly metastatic colorectal cancer line Lim6 and its poorly metastatic parental line LS174T [24], using a similar human antibody array for detection of 500 secreted proteins. Only four secreted proteins; Glypican 3, SLPI, MIP2 and TIMP-1, were up-regulated in highly metastatic Lim6 compared to non-metastatic LSI74T conditioned media. We thus found that SPLI was the only secreted factor increased in metastatic variants of both breast and colorectal cancer cells.

To investigate which, if any, of the three proteins secreted by the metastatic breast cancer cells (SLPI, TSLP, and G-CSF) have a functional role in tumor growth and metastasis, we next used lentivirally-transduced shRNA to stably inhibit translation of SLPI, TSLP, or G-CSF in 4T1 cells. The parental or modified 4T1 cells were then injected into the mammary fat pad of BALB/c mice. When the implanted tumors reached approximately 6 mm in diameter, they were resected and the numbers of lung metastases were assessed 21 days later. We found that down-regulation of G-CSF and TLSP had no significant effect on metastasis, but mice bearing 4T1 SLPI shRNA tumors (with SLPI expression ∼10% of control cell levels measured in 4T1 cells) had significantly fewer lung metastases compared to the control group (Figure 1B and 1C). These results suggest that high expression of SLPI in such breast cancer cells promotes lung metastasis.

### SLPI expression levels correlate with incidence of basal/TNBC metastasis in patients

**Figure 2:**
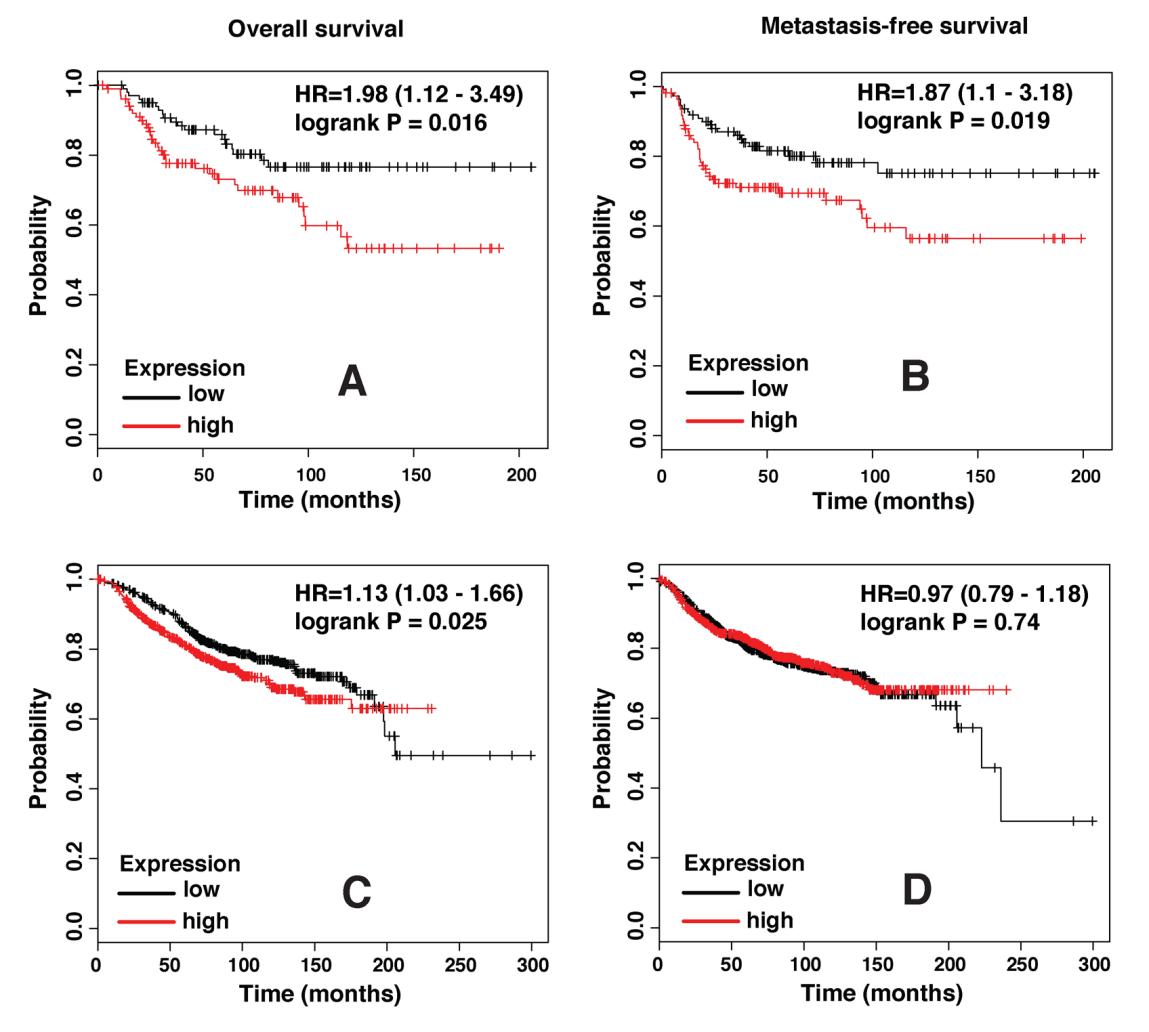
SLPI expression levels strongly correlate with poor outcome in triple negative/basal-like subtype of breast cancer **A.** Plots show overall survival (*n* = 204), and **B.** distant metastasis-free survival (*n* = 229) in basal breast cancer patients with low (black trace) or high (red trace) levels of SLPI expression based on the median value. **C.** Overall survival (*n* = 1117) and **D.** distant metastasis-free survival (*n* = 1609) in all subgroups of breast cancer patients with low or high SLPI expression. HR, hazard ratio.

To determine whether SLPI expression levels correlate with clinical outcome in breast cancer patients, we used the Kaplan-Meier Plotter, a freely available online tool, for survival analysis of data collected in its database. Both distant metastasis-free survival and overall survival were found to be significantly worse in the Basal/TNBC subtype of breast cancer patients with higher SLPI expression in tumor samples (Figure 2A and 2B). Analysis that included all breast cancer patients regardless of their intrinsic subtype also revealed a slightly better overall survival of patients with lower SLPI levels, but there was no significant difference in their distant metastasis-free survival (Figure 2C and 2D). Thus, these clinical data are consistent with our experimental results and support the hypothesis that SLPI regulates metastasis in TNBCs.

### Selection of compound(s) suppressing SLPI expression

**Figure 3:**
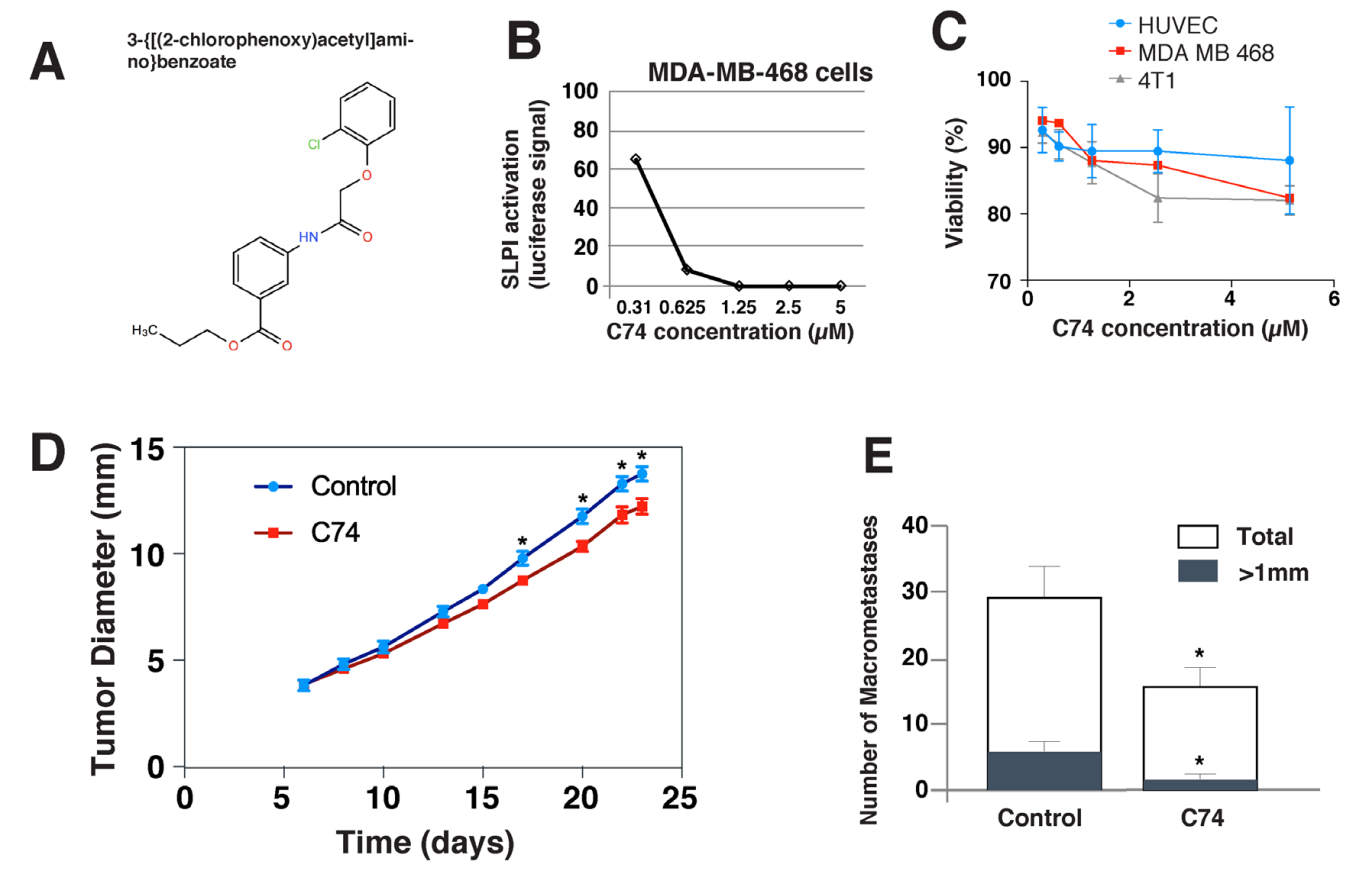
*In vitro* effects of the anti-SLPI compound C74 and its effects on the metastasis and primary tumors *in vivo* **A.** The molecular structure of the selected compound C74. **B.** C74 inhibition of SLPI expression by MDA-MB-468 cells. Cells were treated with C74 (0.31 to 5 µM) for 24 hours, and SLPI expression was quantified using luciferase activity driven by the SLPI promoter. **C.** Viability of HUVEC, MDA-MB-468 and 4T1 cancer cells treated with C74 for 24 hours evaluated using a MTT assay. **D.**, **E.** Anti-SLPI compound C74 suppressed tumor growth and metastasis of 4T1 TNBC in mice. (D) 4T1 cells were injected in the mammary fat pad of female BALB/c mice and the mice were treated with C74 (25 mg/kg) daily for 12 days (from day 6 to day 17) via intraperitoneal injection. Error bars represent SEM; *n* = 8 mice/group, * *P* < 0.05. (E) Quantification of the numbers of large (>1 mm, black bar) and total lung metastases (white bar) observed after harvesting lungs on day 23. Error bars represent SEM; *n* = 8 mice/group, * *P* < 0.05.

After establishing that SLPI is a valid target for inhibiting TNBC metastasis, we next searched for small molecules that we could use to inhibit SLPI pharmacologically. We used the human TNBC cell line MDA-MB-468 with a Luciferase plasmid driven by the SLPI promoter to perform high-throughput screening of a small-molecule library of 60, 000 chemically-diverse compounds. The objective was to identify new biologically active small molecules that were able to reduce SLPI expression in cancer cells by at least 90% without apparent cytotoxicity. We selected 176 compounds that produced > 90% Luciferase inhibition at a concentration of 5 μM. We then confirmed the dose dependence of SLPI repression for the candidate compounds at five different concentrations. Figure 3A demonstrates the results of such an experiment for the eventually selected, best compound C74 (propyl 3-{[(2-chlorophenoxy)acetyl]amino}benzoate). To remove toxic compounds, we chose only 94 of 176 compounds that had no effect on cell viability and proliferation in a MTT assay. The compound C74 has no toxic effect on MDA-MB-468 and 4T1 cancer cells as well as to normal HUVEC (Figure 3C).

### SLPI-repressing compound C74 has anti-metastatic effects

Next, we tested whether treatment with the selected SLPI inhibitor C74 would decrease metastasis *in vivo*. We again used the orthotopic murine 4T1 TNBC model in female BALB/c that metastasizes to the lung, but this time did not resect the primary tumor to assess the effects of C74 on the primary tumor growth as well as dissemination to the lung. The mice were treated with C74 (or DMSO plus PBS as a vehicle) for 12 consecutive days via daily intraperitoneal injections beginning on day 6 after implantation, when tumors were ∼ 3.8 mm in diameter on average (Figure 3D). During the 12-day treatment with C74, there was a small, progressive inhibition of primary tumor growth, which eventually resulted in a statistically significant difference in tumor diameters between the groups. This difference was sustained after therapy stopped. C74 did not cause changes in mouse weight, appearance or behavior. Metastases in the lungs were evaluated after sacrifice of the mice on day 23 (Figure 3E). The total number of metastases in the treatment group decreased by ∼ 50%, and the metastatic colonies were much smaller.

### SLPI displaces RB from FoxM1 and promotes FoxM1 transcriptional activity

**Figure 4:**
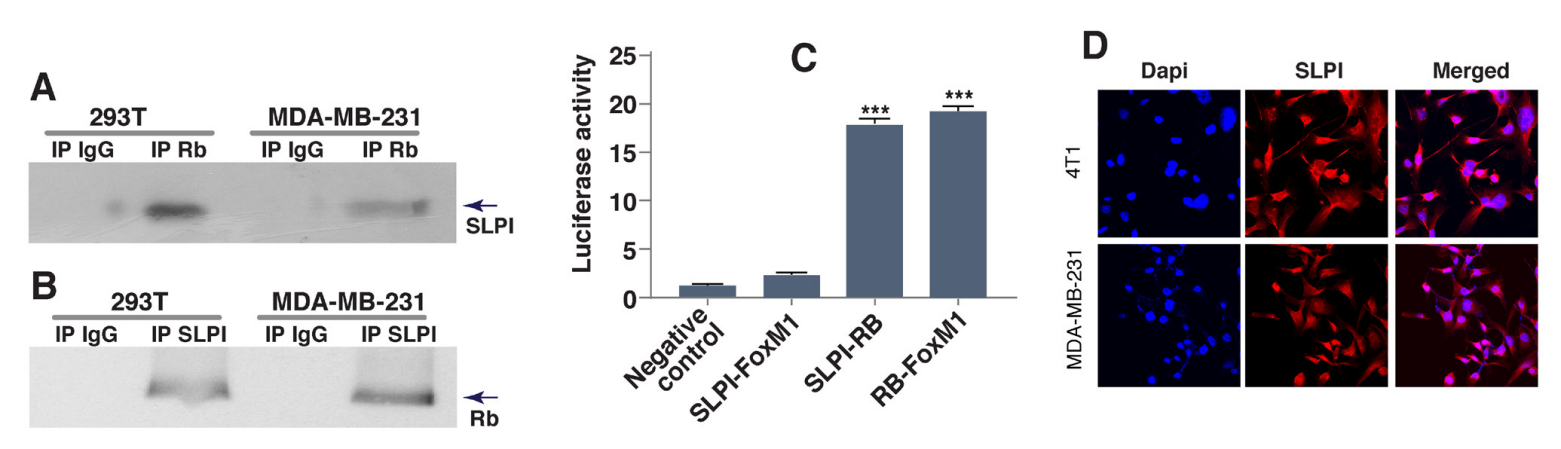
SLPI physically interacts with Rb **A.**, **B.** Co-immunoprecipitation of SLPI and Rb. Total cell lysates from two different cell lines (MDA-MB-231 and HEK293T) were immunoprecipitated and analyzed by immunoblotting with antibodies against SLPI and Rb. Irrelevant isotype-matched IgG antibody was use as a control. **C.** Interaction between SLPI and Rb assessed by mammalian two-hybrid system. We cloned Rb into pBIND, and SLPI and FoxM1 into pACT vectors to generate fusion proteins. Forty-eight hours after transfection the cells were lysed and the amount of luciferases were quantitated using the Dual-Luciferase Reporter Assay System. pACT-FoxM1 and pBIND-Rb were used as a positive control for established physical interaction. Error bars represent SEM; *n* = 3, * *P* < 0.01. **D.** Localization of SLPI protein in breast cancer cells. The images were taken using an Olympus U-TBI90 microscope with 60x Objective. The images show immunostaining with anti-SLPI antibodies (red) and DAPI (blue).

SLPI promoted migration, invasion and metastasis of gastric cancer cells by increasing expression of MMP-2 and MMP-9 genes [25], and the latter could have been targeted by transcriptional factor FoxM1 [26-28]. Furthermore, activation of FoxM1 signaling is generally associated with cancer cellular proliferation, invasion and metastasis [20, 22], and FoxM1 is overexpressed in basal/TNBC [22]. Based on this, we hypothesized that SLPI either directly targets FoxM1, or affects proteins that physically interact with FoxM1 [29]. To test this, we performed co-immunoprecipitation experiments with FoxM1 and its binding partners including tumor suppressor Rb [20, 22, 30]. To determine whether SLPI interacts with Rb, we used protein extracts from two different breast cancer cell lines. Antibody against Rb was used to precipitate Rb complexes and Western blotting with anti-SLPI antibody monitored the presence of SLPI protein in precipitates. SLPI co-immunoprecipitated with Rb, but not with normal rabbit immunoglobulin (IgG), indicating that there is a physical association between these two proteins (Figure 4A). This interaction was further confirmed by the reverse experiment, in which anti-SLPI antibody was used for immunoprecipitation and anti-Rb antibody was used for detection (Figure 4B).

For further verification of this interaction, we used a standard mammalian two-hybrid approach. The two genes encoding two of the three potentially interactive proteins of interest (Rb, SLPI and FoxM1) were cloned into pBIND and pACT vectors to generate fusion proteins with the DNA-binding domain of GAL4 and the activation domain of VP16. HEK293T cells were transiently transfected with a pG5*luc* luciferase reporter plasmid, pGAL4 and pVP16 fusion constructs. After two days, the transfected cells were lysed and the levels of luciferase were quantitated. Interaction between the two test proteins results in an increase in luciferase expression over the negative controls that included pBIND and pACT vectors. The pairs of fusion proteins GAL4-Rb plus VP16-SLPI induced activation of the reporter construct about 7 times more than the negative controls. (Figure 4C). Thus our results with the mammalian two-hybrid system indicate that SLPI protein physically interacted with Rb tumor suppressor but not FoxM1, and also confirmed the known interaction of Rb and FoxM1 proteins.

To confirm that SLPI could also be detected in the nucleus of cancer cells, we immunostained breast cancer cells with anti-SLPI antibody (Figure 4D). High-magnification confocal microscopy revealed that SLPI protein was present not only in the cytoplasm but also in the nucleus of TNBCs. This suggests that SLPI is available to bind nuclear Rb protein, potentially contributing to its regulation.

**Figure 5:**
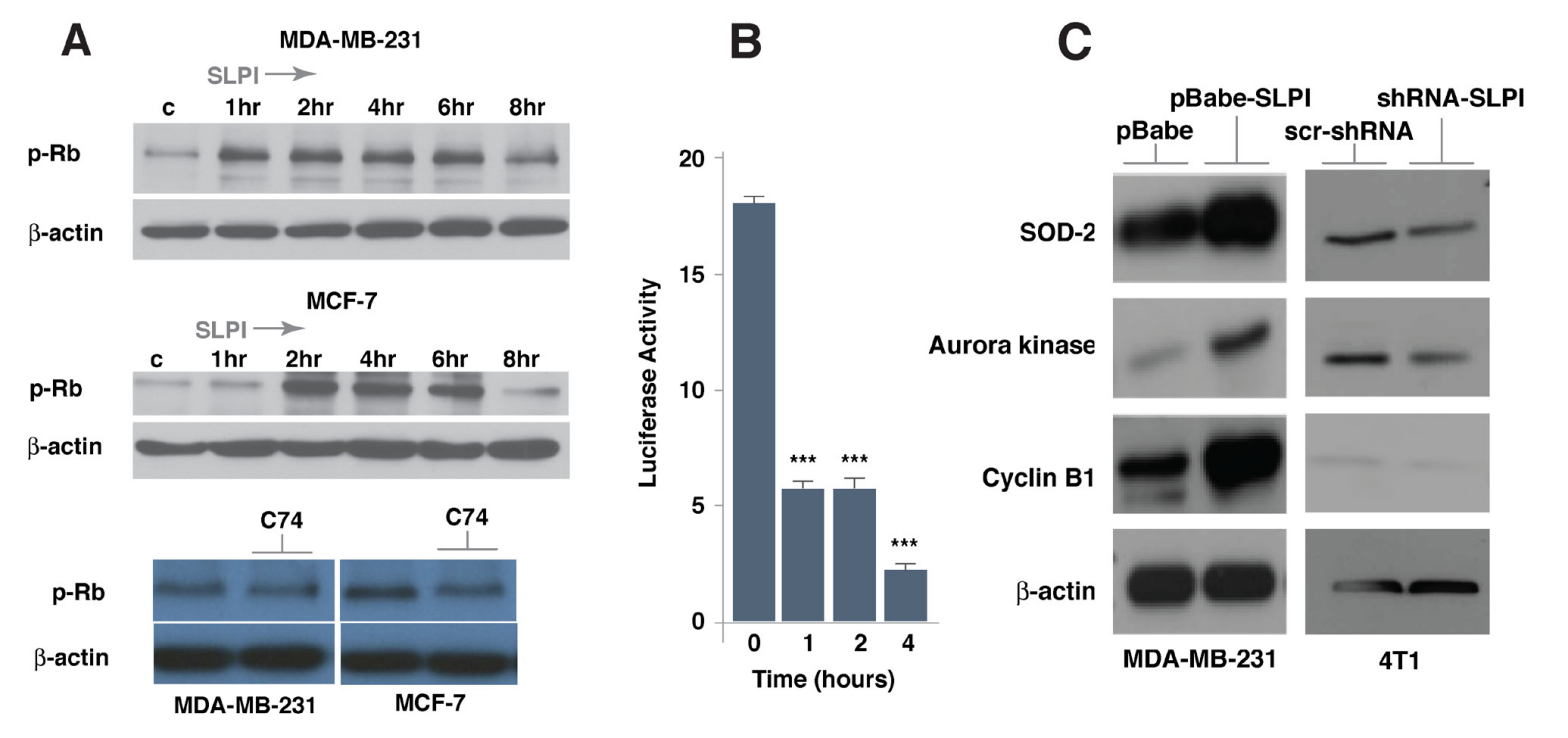
SLPI increases Rb phosphorylation and activates FoxM1 target genes in breast cancer cells **A.** SLPI activated Rb phosphorylation within 1-2 h. MD-MB-231 and MCF7 cells were exposed to recombinant SLPI protein (1.5 µg/mL); proteins obtained from these cells were subjected to gel electrophoresis and Western Blot analysis using antibodies directed against phospho-Rb and β-actin as a loading control. In a time dependent manner, SLPI activates Rb phosphorylation. Compound C74 repressed Rb phosphorylation. MD-MB-231 and MCF7 cells were treated with 1 µM of C74 for 16 h. **B.** The dynamics of SLPI-induced disruption of the physical interaction between Rb and FoxM1. Using a mammalian two-hybrid system with a pair of pBIND-Rb and pACT-FoxM1 fusion proteins with HEK293T cells, we measured the dynamics of binding of Rb to FoxM1 following recombinant SLPI protein treatment. The SLPI was added to cell media in concentration of 1.5 µg/mL. **C.**. Expression of FoxM1 target genes correlate with SLPI level. Western blotting was performed with lysed proteins from MDA-MB-231 cells overexpressing SLPI (pBabe-SLPI), compared to control (pBabe). SLPI was silenced in 4T1 cells using shRNA SLPI lentiviruses and compared to control cells with scr-shRNA lentiviruses. FoxM1 target genes - cyclin B1, aurora kinase B and superoxide dismutase-2 (SOD-2) - were up-regulated in cells with SLPI overexpression and down-regulated if SLPI was suppressed by shRNA.

We then treated several cell lines with recombinant SLPI protein and analyzed the phosphorylation status of Rb (Figure 5A); there was increased phosphorylation of Rb as early as 1-2 h post treatment and this phosphorylation pattern persisted for a few hours. On the other hand, treatment with compound C74 decreased phosphorylation of Rb in breast cancer cells (Figure 5A). Since it had been shown that phosphorylation of Rb leads to its release from FoxM1 binding [29, 30], we then used the mammalian two-hybrid system to confirm the effect of SLPI on the dynamics of the Rb-FoxM1 interaction. We treated cells containing a pair of GAL4-Rb and VP16-FoxM1 fusion proteins with SLPI recombinant protein and demonstrated a progressive decrease of reporter construct activation (Figure 5B). The dynamics of this process after SLPI treatment was similar to that of the Rb phosphorylation (Figure 5A) thus strongly suggesting the interconnection between these two events.

To identify FoxM1-dependent molecular events occurring with modified SLPI expression, we overexpressed the latter protein in MDA-MB-231 cells and performed a series of immunoblotting experiments. We discovered that overexpression of SLPI lead to increased levels of known FoxM1 target proteins, such as cyclin B1, aurora kinase B and superoxide dismutase-2 (SOD-2) (Figure 5C). To confirm this dependence, 4T1 cells were infected with SLPI shRNA lentiviruses. Down-regulation of the SLPI protein decreases expression of these FoxM1 target genes (Figure 5C). Our results collectively show that the level of SLPI protein correlates with FoxM1 transcriptional activity.

### SLPI regulates binding of FoxM1 to its target gene MMP2 and increases cancer cell transmigration through the endothelial layer

**Figure 6:**
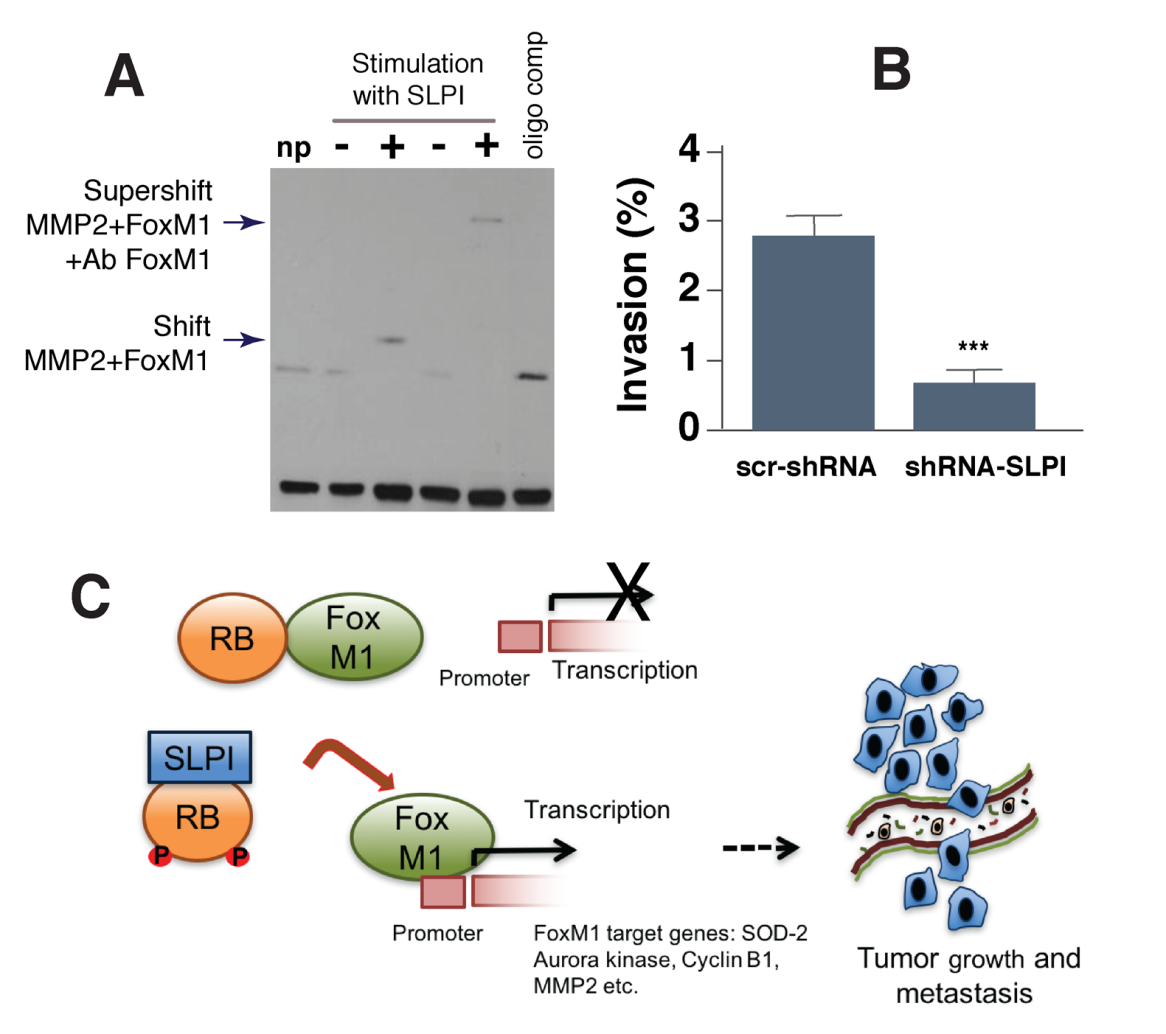
SLPI regulates binding of FoxM1 to its target MMP2 gene and increases cancer cell transmigration through an endothelial cell layer **A.** SLPI regulated FoxM1 binding to the MMP2 promoter sequence in gel shift assay (EMSA). All lanes contained biotin end-labeled MMP2 duplex DNA. NP (lane 1) is a control without protein. Lanes 2 and 3 contained protein extract without and with addition of SLPI protein. Lane 4: non stimulated with SLPI protein extract. Lane 5: supeshift with cells stimulated by SLPI and protein extract was incubated with FoxM1 antibodies. Arrows indicate bands shifted and supershifted by antibodies against FoxM1. Lane 6: the loss of FoxM1-specific DNA-binding in the presence of FoxM1 oligo competitor. **B.** SLPI increases transmigration of breast cancer cells through an endothelial layer in a transwell assay. 4T1 cells with down-regulated SLPI (SLPI shRNA) had decreased transmigration across an endothelial monolayer compared to control 4T1 cells. Error bars represent SEM; *n* = 10, * *P* < 0.001. **C.** Schematic representation of SLPI’s effects on the regulation of the Rb/FoxM1 complex. In the presence of low levels of SLPI, Rb protein binds to FoxM1 and represses the activity of this transcriptional factor. Inhibition of FoxM1 leads to repression of FoxM1 target genes that are associated with tumor growth and metastasis. In cancer cells that express high levels of SLPI, it physically interacts with Rb to facilitate Rb release from FoxM1. This makes FoxM1 available to initiate transcription of target genes involved in tumor growth and metastasis.

To directly test the ability of SLPI to modify FoxM1 binding to another target gene, MMP2, we used the electrophoretic mobility shift assay. The MMP2 promoter sequence comprising the FoxM1-binding site (-662 to -671 bp) was biotin-labeled and used as a probe for the protein binding gene promoter region [26]. The probe was incubated with protein extracts from SLPI-treated MDA-MB-231 cells and untreated cells. This experiment demonstrated (Figure 6A) that SLPI induced protein binding to the probe, as reported by the retardation of its mobility (shift of bands in lane 3 vs. lane 2). Thus treatment of cells with SLPI resulted in a substantial increase in binding activity of FoxM1. The binding of this protein to the probe was sequence-specific, as it was blocked by competition with an unlabeled FoxM1 oligomer. To confirm that FoxM1 indeed bound to MMP2 promoter in the presence of SLPI, we carried out a supershift assay by adding FoxM1-binding antibody to that protein-DNA complex. The enhanced probe retardation is shown in lane 5 (Figure 6A). This assay showed that FoxM1 binds to the MMP2 promoter and that SLPI stimulates FoxM1 binding activity (Figure 6A).

To determine whether SLPI promotes metastasis with enhancing vasoinvasive properties of cancer cells, we used a transwell cell migration and invasion assay [31]. We plated either 4T1-GFP control or 4T1-GFP shSLPI cells onto confluent endothelial monolayers growing on a transwell insert and in 24 h quantified the number of such GFP-labeled cancer cells that invaded through the endothelial layer to the bottom of the insert. We found that shSLPI-treated 4T1 cells exhibited a significant reduction in their invasive capability despite no change in the rate of proliferation (Figure 6B). These data indicate that the vasoinvasiveness of 4T1 cancer cells was at least partially dependent on SLPI signaling.

## DISCUSSION

Proteins secreted from tumor cells may govern cancer aggressiveness and metastasis via various, often unknown, interactions within the host. SLPI is known to be of significant prognostic and/or predictive value for lung, gastric and colorectal carcinomas [10-13] but its role in breast cancers is somewhat less clear. In animal studies, overexpression of SLPI in implanted breast cancer cells typically lead to enhanced local growth and increased metastasis [17-19], but the opposite effect at the primary site has also been reported [32]. In the clinic, gene expression analysis for breast cancer patients revealed that high SLPI was associated with a shorter time to tumor relapse and shorter overall survival [16], while the opposite correlation was reported for time to relapse using another dataset [33]; moreover, the levels and the role of SLPI expression in different breast cancer subtypes have not yet been systematically compared.

In this study, we identify SLPI as an important pro-metastatic component of the secretome for breast cancers, primarily for TNBCs. We first found over-secretion of SLPI in metastatic murine 4T1 TNBC, compared to its non-metastatic counterpart, 67NR. We then demonstrated a decrease of lung metastasis from orthotopically implanted 4T1 tumors if SLPI production was down-regulated in the tumor cells using shRNA. Of note, although SLPI turned out to be the only over-secreted protein with pro-metastatic activity *in-vivo*, it is possible that the array used for protein detection lacked that resolution to identify other potentially important candidate molecules.

Since SLPI was involved in development metastasis in TNBC animal model, we then analyzed the available clinical database. This analysis showed that higher SLPI expression (mRNA level) in tumor samples is associated with shorter overall survival of patients with breast cancer in general, as well as in the subset with basal/TNBC. Metastasis-free survival correlated with SLPI only in the basal/TNBC cohort. Finally, we identified a compound in a small-molecule library that inhibited SLPI at non-toxic concentrations and pharmacologically decreased both primary 4T1 tumor growth and lung metastasis. Taken together, these results support that SLPI is a central regulator of dissemination and colonization of TNBCs.

The precise mechanisms by which SLPI enhances cancer aggressiveness and metastasis are not well understood. In this study, we provide two new pieces of information. The first is related to how SLPI affects the interaction of cancer and endothelial cells. The role of SLPI has already been associated with specific features of primary tumor vascularization and vascular mimicry in the sites of metastasis [18, 34]. In our in-vitro experiment we show here that SLPI secreted by breast cancer cells significantly enhances, in a paracrine manner, their transmigration through an endothelial cell monolayer, implying a similar pro-metastatic intravasation effect *in-vivo*.

The second mechanism discovered, although not directly determined by SLPI secretion from tumor cells, is clearly connected with intracellular SLPI production, which operates via FoxM1 intracellular regulation to alter the expression of other secretory proteins with pro-metastatic activity. The FoxM1 activity is inhibited by several mechanisms including auto-repression by its inhibitory N-terminal region and interaction with its inhibitors - Rb and p19Arf [22, 23, 29, 30]. The Cyclin D1/Cdk4 can phosphorylate Rb leading to a disruption of Rb-FoxM1 complex, which subsequently activates FoxM1 [30]. Our results indicate that SLPI has a similar influence on the Rb protein. Importantly for this interaction, we detected SLPI inside the nucleus of cancer cells. Also, overexpression of the SLPI in gastric cancer cells increased the expression of FoxM1 target genes iMMP-2 and MMP-9 and promoted the migration of cancer cells by degrading collagen [3, 25] but FoxM1 was not examined. Our results on breast cancer cells are in line with that observations and directly link SLPI expression to regulation of FoxM1 function. Figure 6C summarizes a potential molecular mechanism of FoxM1 activation through the interaction of Rb with SLPI.

In conclusion, we have identified SLPI as a new target for anti-metastatic therapies and provided insights into the molecular mechanisms of FoxM1 regulation.

## MATERIALS AND METHODS

### Cancer cells and their modifications

Human triple-negative MDA-MB-231, LS174T, HEK293T and MCF-7 and MDA-MB 468 cell lines were obtained from the ATCC collection. MDA-MB-468 cells stably expressing luciferase plasmid driven by the SLPI promoter were used for high-throughput screening. To create SLPI overexpressing cells, we infected cancer cells with pBabe-SLPI retroviruses containing the LTR promoter and puromycin resistant genes. The SLPI knockdown 4T1 cell line was established by lentiviral infection of 4T1 cells. Lentiviral particles were produced by transfecting 293T cells with two different shRNA clones that targeted SLPI (MISSION shRNA Sigma) and viral packages (VSVG, psPAX2) using FuGENE reagent (Promega, US). SLPI down-regulation was confirmed by using both q-PCR and ELISA.

### Detection of the expression levels of secreted proteins

Mouse and human antibody arrays (RayBiotech, Inc, US) were used for detection of proteins in cell culture supernatant. The expression levels of 308 mouse and 507 human target proteins were simultaneously detected, including cytokines, chemokines and other secreted proteins. The biotin-labeled samples were added onto array, incubated with HRP-streptavidin and subsequently visualized by chemiluminescence according to manufacturer protocol.

### Compound library and high-throughput screening

We used a compound library from the Small Molecule Screening Core of Roswell Park Laboratory for the high-throughput assay. This library includes 60, 000 compounds from different sources: 1) FDA-approved drugs from Prestwick, 2) purified natural products, and 3) small molecules purchased from Chembridge, Maybridge, CEREK and Bionet Research Ltd. For the high-throughput screen (HTS), the 5 µM compounds were diluted in 100% DMSO, and spotted per well to 384-well plates using a Multimek 96/384 Channel Automated Pipetter. The average molecular weight of the compounds in the library is 400 Da (range=225-600), and a large proportion of this library has been tested successfully for a number of screening assays. We used a cell-based readout system for compound selection. MDA-MB-468 cells stably expressing luciferase plasmid driven by the 251 bp SLPI promoter were used for high-throughput screening. For our cell-based screening, we selected an optimal cell density of 10, 000 breast cancer cells/well to produce the most prominent signal. MDA-MB-468 cells were treated with 5 μM of compound for 24 hours. We further studied selected compounds with 90% of SLPI repression. We confirmed the specificity of SLPI repression for the candidate compounds at five different concentrations (0.31 µM, 0.625 µM, 1.25 µM, 2.5 µM and 5 µM). We eliminated toxic compounds from selected groups by analyzing treatment effects on normal human umbilical vein endothelial cells (HUVEC).

### Assessment of spontaneous lung metastases

All mouse experiments were approved by the Massachusetts General Hospital Institutional Animal Care and Use Committee and were performed in accordance with NIH guidelines. The OPRR Animal Welfare Assurance number is A3596-01, 9/17/97. Parental and modified 4T1 cells were injected into the third mammary fat pad of 8-week-old female BALB/C mice, 10^5^ cells in 20 μl of HANKS per injection. The growing tumors were then either resected at a certain size or left as is. In the latter case, the mice were treated daily with i.p. delivery of 20 mg/kg of the selected compound suppressing SLPI (or vehicle), for 12 consecutive days starting from day 6 after tumor implantation. The mice were sacrificed either on day 21 post primary tumor resection or 17 days after compound treatment initiation. The lungs were removed and placed in Bouin’s solution (Sigma). A few days later, the lung surface was examined under a stereomicroscope and the number and size of metastases were evaluated.

### Patient survival analysis

The Kaplan-Meier Plotter (www.kmplot.com and [35] was used to compute Kaplan-Meier plots for SLPI in basal intrinsic subtype and for all breast cancer patients. We analyzed a database of 2014 patients and correlated Affymetrix microarray results for SLPI (measured in patient tumor samples) with associated survival information, with a mean follow-up of 69 months. The Plotter automatically determined the median-based cut-off used to split the patients into “high SLPI” and “low SLPI” expression categories. The probe 203021_at was selected for SLPI. Analysis was performed using default parameters and the exclusion of outlier arrays from the array quality control tab.

### Western blotting and immunoprecipitation

Lysates from 293T, MDA-MB-231, MCF7 and 4T1 cells were prepared in lysis buffer containing Tris-HCl 50 mM/pH 7.4; NaCl 150 mM; NP-40 1%; SDS 0.1%; Na-deoxycholate 0.5%; EDTA 1 mM; plus 1% phosphatase inhibitor cocktails I and II (Sigma-Aldrich, US), and 1% protease inhibitor cocktail (Roche). Equal amounts of protein (50 μg/sample) were resolved by SDS-PAGE, transferred onto nitrocellulose membranes, and immunoblotted using the following primary antibodies: anti-SLPI, anti-RB (Cell Signaling and Santa Cruz Biotechnology, US), anti-SOD2, Aurora kinase, cyclin B1 and anti-β-catenin (Cell Signaling Technology). For co-IP assays, lysates were pre-cleared with IgG, and incubated with anti-SLPI, anti-RB or control IgG antibodies linked to Sepharose-A beads. Protein complexes were dissociated from beads and separated by SDS-PAGE. Immunodetection was performed by incubation with HRP-conjugated species-specific antibodies, followed by chemiluminescence detection (Perkin Elmer, US).

### Immunohistochemistry and imaging

Breast cancer cells were grown in 8 wells slide chambers coated with 0.1% gelatin. Once the cells form a confluent monolayer, the cells were fixed and stained for SLPI with SLPI antibodies (Sigma). Stained cells were imaged using Olympus IX81 scanning confocal microscope with 60X 1.35NA oil immersion UplanSApo lens.

### *In vitro* invasion assay

Fluoroblok transwell inserts (8 μm pore, Falcon) were coated with 0.1% gelatin (Sigma) before seeding 1 x 10^5^ human umbilical vein endothelial cells (HUVEC) onto the polystyrene membrane. 4T1-GFP tumor cells that seeded on and invaded through the endothelial monolayer were analyzed in 24 hrs to quantify the percent of invading cells*.*

### Electrophoretic mobility shift assay

We carried out electrophoretic mobility shift assays (EMSAs) following manufacture protocol using kit from Thermo Fisher. We used double-stranded oligonucleotides from MMP2 promoter with FoxM1 binding site: 5’-CTGTTCAAGATGGAGTCGCTCTGGTTC-3’. End-labelled DNA was detected using streptavidin-conjugated horseradish peroxidase. For the supershift assay, antibody against FoxM1 (Cell Signaling) was added to the binding reaction for 1 hour on ice.

### Statistical analysis

All data are expressed as mean ± SEM. For statistical analysis, JMP Statistical analysis software was used (SAS Institute, NC). Two-tailed t tests were used to compare data between two groups. We considered a *p*-value less than 0.05 to be statistically significant.

## Abbreviations

SLPI: Secretory leukocyte protease inhibitor
TNBC: triple-negative breast cancer
Rb: retinoblastoma tumor suppressor protein
FoxM1: Forkhead Box M1
SOD-2: superoxide dismutase-2
